# *Trypanosoma cruzi* in Bats (Chiroptera; Mammalia) from the Brazilian Atlantic Forest, São Paulo State

**DOI:** 10.3390/microorganisms12050945

**Published:** 2024-05-07

**Authors:** Danilo Alves de França, Mariana Louro, Sara Zúquete, Dayane da Silva Zanini, Gustavo Nunes de Moraes, Gabrielle dos Santos Rocha, Leandro Meneguelli Biondo, Felipe Fornazari, Benedito Donizete Menozzi, Isabel Pereira da Fonseca, Helio Langoni

**Affiliations:** 1 Department of Animal Production and Preventive Veterinary Medicine, School of Veterinary Medicine and Animals Science, São Paulo State University, Botucatu 18618-681, SP, Brazil; danilo.franca@unesp.br (D.A.d.F.); f.fornazari@unesp.br (F.F.);; 2 Department of Animal Health, Faculty of Veterinary Medicine, University of Lisbon, Avenida da Universidade Técnica, 1300-477 Lisboa, Portugalifonseca@fmv.ulisboa.pt (I.P.d.F.); 3 CIISA—Centre for Interdisciplinary Research in Animal Health, Associate Laboratory for Animal and Veterinary Sciences (AL4AnimalS), Faculty of Veterinary Medicine, 1300-477 Lisbon, Portugal; 4 National Institute of the Atlantic Forest (INMA), Brazilian Ministry of Science, Technology and Innovation, Santa Teresa 29650-000, ES, Brazil; 5 Interdisciplinary Graduate Studies, University of British Columbia, Kelowna, BC V1V 1V7, Canada

**Keywords:** emerging disease, vampire bats, urban bats, molecular epidemiology, PCR, phylogeny

## Abstract

The causative agent of Chagas disease is *Trypanosoma cruzi*, which is widely distributed throughout the South American continent and extends into North America. Its occurrence in bats is poorly described and may impact the disease’s maintenance and epidemiology. The aim of this study was to detect the agent by PCR assays targeting kDNA and nuclear DNA in the organs of 203 urban bats and rural vampire bats from the Brazilian Atlantic Forest, São Paulo state, during the pandemic period from 2020 to 2022. In total, 6 of the 203 bats (2.97%) were positive for *T. cruzi*. Infection was detected in 2% (2/101) of *Desmodus rotundus*, 33% (1/3) of *Nyctinomops laticaudatus*, 25% (1/4) of *Artibeus lituratus*, 4% (1/24) of *Eumops glaucinus* and in 2% (1/41) of *Molossus molossus*. The gene sequences obtained were assessed for quality and deposited in a public repository. Fruit bats were statistically associated with positivity for *T. cruzi*. To our knowledge, this study detected *T. cruzi* for the first time in bats from São Paulo state and in *N. laticaudatus* and *E. glaucinus* species.

## 1. Introduction

Around sixty percent of emerging infectious diseases affecting humans are zoonotic, and more than two thirds of them originate in wildlife [1]. Microorganisms often hide in reservoir hosts, sheltering them chronically while they suffer little or no illness. When an ecological disturbance occurs, these agents reveal themselves [2]. Experts say that in the last fifty years, the number of emerging diseases has increased fourfold, mainly due to the constant expansion of areas inhabited by humans into animal habitats, especially in tropical regions identified as hotspots for the spread of diseases around the world [3].

Bats, due to their wide genetic diversity, can be a favorable environment for the mutation and evolution of pathogens, and these genetic changes can give rise to more virulent or more transmissible variants that pose a threat to global health [4]. The detection of diseases in bats can serve as an early warning indicator of the presence of dangerous pathogens. This can provide valuable information for preventing and controlling infectious disease outbreaks in humans. In addition to human invasion of their natural habitats, ecological imbalance and the process of migration in search of food also favor the spread of pathogens [4].

Chagas disease is a disease characteristic of the American continent and is the result of infection caused by the protozoan *Trypanosoma cruzi*. The vectors of *T. cruzi* are *Triatoma infestans*, *Rhodnius prolixus*, and *Triatoma dimidiata*, all widely distributed throughout South America. The presence of infected reduviids extends into North America [5].

Triatomines are widely present in homes and peridomiciles, as well as in caves and shelters, and can have close contact with bats, mainly by feeding on these bats, in the case of insectivorous bats. There are currently around 10 million people infected, 25 million people at risk of contracting the infection and around 20,000 deaths a year from the disease [5]. In Brazil, the largest country in South America, 1570 cases of the disease were recorded by health agencies between 2000 and 2013. In the state of São Paulo, there were only four confirmed cases during the entire period [6].

In 2018, a Brazilian study in the state of Acre, in the Amazon region, analyzed 367 bats of different species, detecting the genus *Trypanosoma* in 81 samples. Of these, 16.5% (60/367) were *T. cruzi* [7]. It is worth noting that Acre, like São Paulo, has had few recorded human cases of Chagas disease in the last decade. Another study in the Amazon region detected 20.3% (15/74) of *T. cruzi* in bat samples [8].

There is a lack of research on Brazilian bat populations. According to our knowledge, no studies on bats have been carried out in the state of São Paulo, Brazil’s most populous region and home to an extensive area of Atlantic Forest, a biome where there is a great diversity of urban bat species and vampire bats in rural areas. In order to understand the dynamics of *T. cruzi* infection in bats in the state of São Paulo and expand research into the role of bats in the epidemiology of Chagas disease, a molecular investigation was carried out using organs of bats of different species and feeding habits from different regions of the state, as well as phylogenetic analysis of the sequenced agents.

## 2. Materials and Methods

### 2.1. Ethics Statement

The Ethics Committee for Animal Use of the School of Veterinary Medicine and Zootechnics, São Paulo State University, Brazil (protocol number 0259/2022) approved this study. We obtained environmental authorization through the Biodiversity Authorization and Information System (SISBIO) from the Chico Mendes Institute for Biodiversity Conservation (ICMBio), protocol number 85973-1.

### 2.2. Study Design and Sampling

A total of 203 bat samples were obtained from São Paulo state, where part of the Brazilian Atlantic Forest is located. There were samples from nine bat genera and fourteen species: *Desmodus rotundus* (n = 101); *Molossus molossus* (n = 41); *Molossus rufus* (n = 2); *Myotis nigricans* (n = 13); *Eumops glaucinus* (n = 24); *Eumops auripendulus* (n = 4); *Eumops perotis* (n = 2); *Eptesicus brasiliensis* (n = 3); *Eptesicus furinalis* (n = 2); *Artibeus lituratus* (n = 4); *Lasiurus blossevillii* (n = 3); *Nyctinomops laticaudatus* (n = 3); *Histiotus velatus* (n = 1).

**Figure 1:**
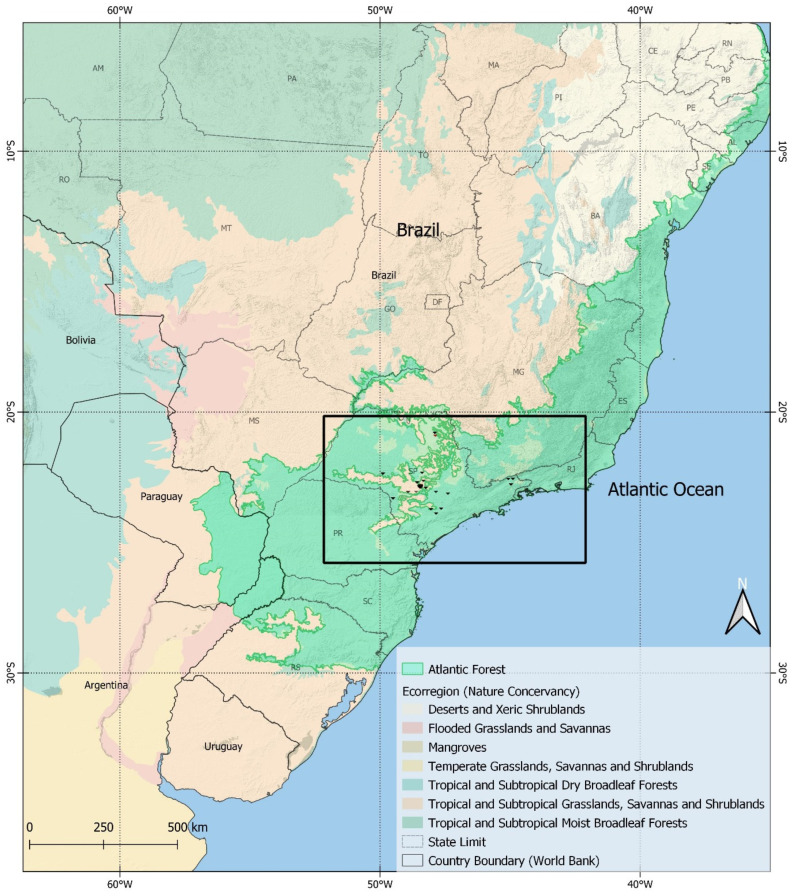
The study area’s map showing the Brazilian Atlantic Forest located in South America and the state of São Paulo inside the square. The bat collection areas are marked with bat flags.

The Atlantic Forest is made up of several forests and ecosystems of its own, with more than 2000 native animal species, including birds, mammals, reptiles, amphibians and fish, covering 15% of Brazil’s territory. More than 70% of the Brazilian population lives in this area, so the biodiversity of the forests is very close to human beings. The forests of this region have suffered from deforestation and human invasion over the last few centuries, and currently, only 7% of the original forest remains [9]. The samples were obtained in the years 2020 to 2022, a pandemic period that resulted in around 37 million confirmed human cases of COVID-19 and 700,000 deaths in Brazil. In the state of São Paulo alone, where this study was carried out, there were more than 6 million confirmed cases and 181,000 deaths, which led to a prioritization of dealing with the disease in the country and a consequent deficiency in the control of other prevalent zoonotic diseases [10]. Figure 1 shows the study area [11,12,13].

The samples of fruit bats and insectivorous bats came from animals that had fallen into people’s homes in urban areas and were sent alive by the municipalities to the regional Zoonosis Diagnostic Service for rabies diagnosis. The samples of vampire bats were obtained in conjunction with the Agricultural Defense Office (EDA), which carries out active rabies surveillance work in the state of São Paulo, aiming to diagnose rabies and monitor cases in the region.

The bats received alive were euthanized with a supra dose of isoflurane inhalation anesthetic soaked in absorbent cotton, in a closed container with a physical separation between the absorbent cotton and the animal, and kept there until the animal stopped moving. After removal of the brain for rabies diagnosis, fragments of the heart, spleen, lungs, liver, intestines and kidneys were collected aseptically for investigation of *T. cruzi*. The animals’ hearts and kidneys were screened, and in positive cases, the other organs were tested. The collected material was stored in DNA-free microtubes at −80 °C. The taxonomic identification of the bat species was made using the criteria described for determining Brazilian chiroptera [14]. Data on the sex and collection site of the animals was also recorded.

### 2.3. DNA Extraction and Quality Assessment

DNA was extracted individually from each animal sample using the Illustra Tissue and Cells Genomic Prep Mini Spin Kit (GE Healthcare Life Sciences, Marlborough, MA, USA), following the manufacturer’s instructions. The purified DNA samples were eluted in 100 μL. The quality of the DNA was assessed by the concentration, which was then adjusted up to 100 ng/µL, and by the absorbance ratios of 260/280 and 260/230 nm using a Nanovue spectrophotometer (GE Healthcare Life Sciences, Chicago, IL, USA).

### 2.4. Trypanosoma cruzi DNA Detection

All DNA samples were submitted to a cPCR assay targeting the *T. cruzi* kinetoplast, using primers 121 (5′ AAA TAA TGT ACG GGT GAG ATG CAT GA 3′) and 122 (5′ GGG TTC GAT TGG GGT TGG TGT 3′) [15]. All positive samples were subjected to a second cPCR reaction targeting *T. cruzi* nuclear DNA, using the primers TCZ1 (5′ CGA GCT CTT GCC CAC ACG GGT GCT 3′) and TCZ2 (5′ CCT CCA AGC AGC GGA TAG TTC AGG 3′) [16]. In both reactions, the final volume was 25 μL and the mixtures contained 2 μL of DNA template, 1.5 mM of MgCl2 (50 mM/1 mL), 200 μM of dNTP mixture, 3.5 U of Taq DNA polymerase, 10 pmol of each primer and the rest of the volume made up of ultrapure water. Sterile milli-Q water was used as a negative control and the products of DNA extraction from strains maintained in NNN-LIT medium, under cultivation in a B.O.D. oven at 25 °C, repeated every two weeks, of *T. cruzi* (“Y” strain) were used as a positive control. The amplifications were performed in a thermal cycler (Applied Biosystems, Foster City, CA, USA).

Reaction first round: denaturation at 94 °C for 3 min, followed by 35 cycles at 94 °C for 30 s, 62 °C for 60 s, 72 °C for 60 s and then a final extension step at 72 °C for 10 min. Reaction second round: denaturation at 94 °C for 30 s, followed by 25 cycles at 94 °C for 30 s, 66 °C for 30 s, 72 °C for 30 s and then a final extension step at 72 °C for 30 min. The products of all the cPCR assays were separated by electrophoresis on 1.5% agarose gel, stained with Sybr Safe (Invitrogen, Waltham, MA, USA) 0.1 µL/mL, in a horizontal vat containing TBE 1× (0.1 M Tris, 0.09 M boric acid and 0.001 M EDTA) and a voltage of 100 V/150 mA for 40 min. For the molecular weight standard, the DNA Ladder 100 bp (Invitrogen, Waltham, MA, USA) was used in order to visually compare the size of the amplified fragments. The gels were visualized under ultraviolet light (Bio-Rad, Hercules, CA, USA) using Image Lab software version 4.1.

### 2.5. Sequencing and Phylogenetic Analyses

The amplified products were purified using the NZYGelpure Kit (NZYTech, Lisbon, Portugal) according to the manufacturer’s instructions and sequenced with 7 μL of the purified sample added to a solution containing 3 μL of each primer (3.2 pmol). Sequencing took place in the forward and reverse directions with the TCZ1 and TCZ2 primers used in PCR. Sequencing took place using the BigDyeTM Terminator v3.1 Cycle Sequencing Kit (Applied Biosystems, Foster City, CA, USA). The sequences obtained from the positive samples were submitted to the BLASTn program for nucleotide analysis in comparison with sequences from the international database (GenBank). The quality of the sequences was analyzed using BioEdit Sequence Alignment Editor summer 7.2. The absence of overlapping peaks, sufficient amount of DNA, smoothness of the peaks and uniform spacing were evaluated for the selection of viable sequences.

A phylogenetic tree was constructed including all the sequences generated in this study together with representative reference sequences from GenBank. South American sequences of *T. cruzi* reference strains that have recently been published were added to build the phylogenetic tree. Phylogenetic analysis was carried out using distance methods in MEGA11 version 11.0.13. The Neighbor-Joining method was applied using Kimura’s two-parameter model. The topologies of the inferred trees were evaluated by 1000 bootstrap resamplings.

### 2.6. Data Analysis

The Chi-square test was used to compare the molecular prevalence of *T. cruzi* for the epidemiological variables. The significance level was 0.05. All tests were performed using SAS Studio version 3.81 (SAS Institute Inc., Cary, NC, USA).

## 3. Results

**Figure 2:**
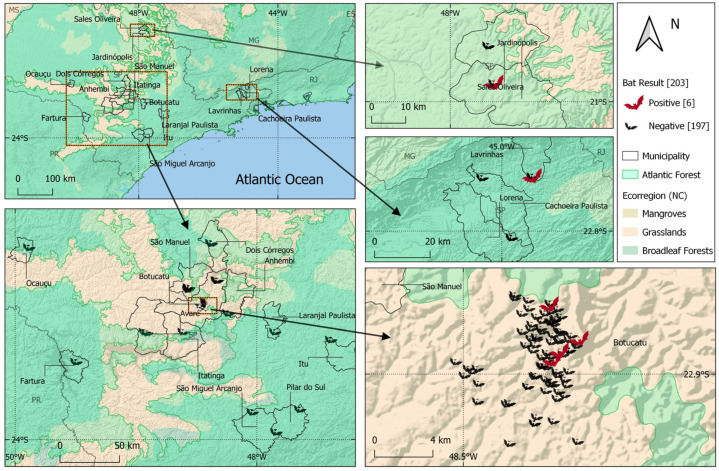
Distribution map of *Trypanosoma cruzi* infection detected in bats from Brazilian Atlantic Forest, São Paulo state (2020–2022).

DNA of *T. cruzi* was amplified in samples from six bats, representing 3% [CI (95%) = 1.4–6.3%] of the total number of samples. Of the positives, there were 33% (1/3) of *N. laticaudatus*, 25% (1/4) of *A. lituratus*, 4% (1/24) of *E. glaucinus*, 2% (1/41) of *M. molossus* and 2% (2/101) of *D. rotundus*. Amplification occurred in the heart samples of four animals, kidney samples of three animals, liver samples of three animals and lung samples of two animals. There was no amplification in the intestine and spleen tissues. Figure 2 shows the distribution of infection in bats according to the study area [11,12,13].

The risk assessment showed that the bats’ feeding habits were statistically associated with *T. cruzi* positivity. Fruit bats were 10.5 and 16.5 times more likely to be positive than insectivorous and hematophagous bats, respectively. For the other variables, there was no statistical difference (Table 1).

The standard sequencing results showed over 97.4% identity with *T. cruzi* sequences in all samples. The sequences obtained were deposited in GenBank: accession numbers OR736031-OR736036. The full results of the BLAST analysis, including score, query coverage and identity percentage, can be found in Table 2.

**Figure 3:**
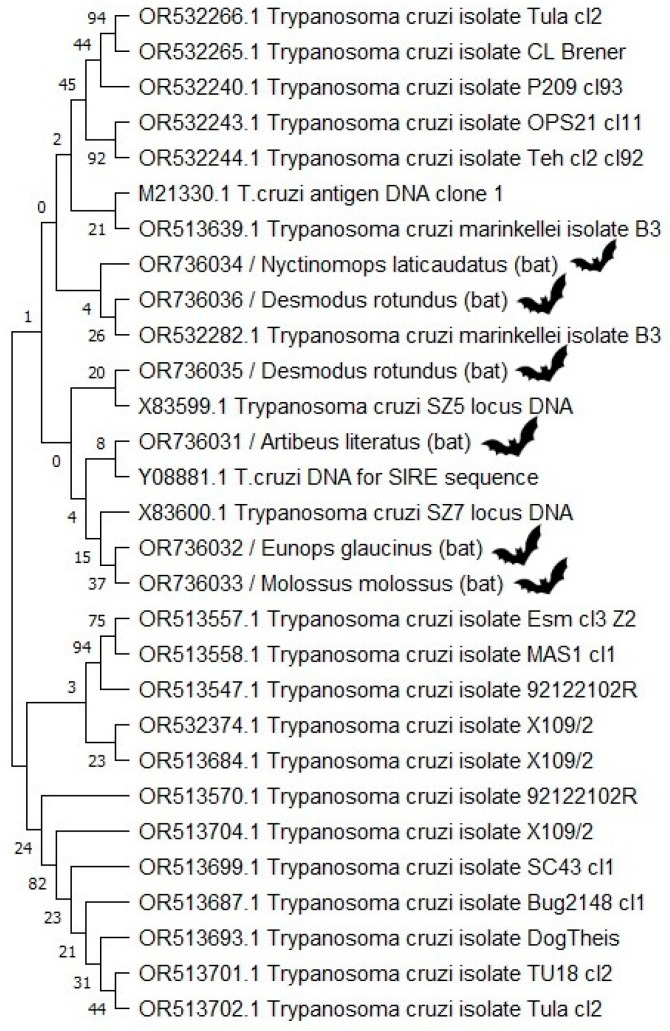
Phylogenetic tree comparing 23 *Trypanosoma cruzi* nuclear DNA reference segments with the sequences generated in this study, marked with bat flags, was built using the Neighbor-Joining method in Mega11.

In the phylogenetic tree shown (Figure 3), represented by the bootstrap consensus tree, we observed a similarity between the sequences OR736036 from *D. rotundus* and OR532282.1 from a *Phyllostomus discolor* bat from São Luís, Bahia. Both sequences were genetically close to the OR736034 sequence from *N. laticaudatus*. The second *D. rotundus* sequence generated in this study, OR736035, was similar to the X83599.1 sequence of the Argentine strain, and had very low proximity to the first *D. rotundus* sequence. The OR736032 and OR736033 from *E. glaucinus* and *M. molossus*, respectively, sequences were genetically similar. The OR736031 sequence from *A. lituratus* was relatively close to Y08881.1, an Argentine isolate from *Mus musculus*, and somewhat close to the two bat sequences mentioned above.

## 4. Discussion

The prevalence found in this study shows that, as elsewhere on the continent, *T. cruzi* has infected vampire and non-vampire bats. The potential for transmission of the agent to other animals and humans is still under discussion and requires targeted studies. A metagenomic study of the saliva of neotropical bats revealed the presence of *T. cruzi* and suggested, together with phylogenetic results, an independent transmission cycle, where transmission through saliva is a possibility. This can occur through biting in the case of vampire bats, consumption of contaminated fruit and licking during bat socialization [17]. In addition, as these animals have contact with flies, mosquitoes and ticks capable of carrying pathogens, although the vectors of Chagas disease are triatomines, the infection of these other ectoparasites has already been proven [18,19].

There are several ways in which these animals can become infected. One of these ways is vertical transmission from mother to fetus, which has already been described in some bat species, and this is strengthened by the fact that bats have been studied as ancient reservoirs of pathogens and the source of various diseases in populations [20]. It is true that the physiology of bats, developed for flight conditions, allows a compatible and favorable blood regulation for the development and maintenance of several families of viruses, and although this characteristic cannot be applied to protozoa, considering that favorable characteristics for the maintenance of certain pathogens can be perpetuated through the generations by vertical transmission is important for the study of infections in bats [21].

Another transmission form is ingesting triatomines or fruit contaminated with triatomine feces containing *T. cruzi* forms. Our study showed a significant association between fruit bats and positivity. In Brazil, the presence of these triatomines in sugar cane plantations is a reality, and so is the presence of fruit bats in these plantations. It is believed that urban bats have a greater potential to transmit agents of diseases than other bats, as they tend to stay in the same place and experience a reduction in regular exposure to microorganisms that they used to acquire in the wild. This previous exposure helped to keep infection to a minimum. As a result, more bats, due to factors such as malnutrition, loss of habitat and other elements, end up being infected and consequently spreading a greater amount of microorganisms in urban areas [22]. There were no statistical differences in our study in relation to location; however, in this specific case, the idea runs counter to the fact that Chagas disease occurs more in rural areas, where there is a greater presence of vectors [6].

Vampire bats do not feed on fruit or insects, and the possible reason for this infection may be contact with triatomine feces in shelters and roosts or on the backs of livestock. Other wild mammals may be related to the presence of *T. cruzi* in bats since the infection has already been described in some of these species prevalent in the Brazilian Atlantic Forest [5]. Transmission through triatomine feces could be a possibility, considering that they usually contaminate sugar cane, a regional plant that is very common in the state and that bats often feed on [5]. Some bats, such as *D. rotundus*, are known to be hematophagous and transmit rabies to livestock and river-dwelling populations. Because they feed exceptionally well on blood, they are much less resistant to fasting, due to the absence of pancreatic β-cells that are characteristic of these types of bats. This absence prevents the bat’s liver from producing glycogen reserves and means that the search for food has to be daily, making their contact with other animals even greater, and also increasing the chances of interaction between animals and new pathogens [23].

The flight abilities of bats influence the occurrence of new diseases not only physiologically, but also geographically, as they are able to fly long distances in search of food, depending on the relief conditions of the region, and consequently act as a migratory agent. Bats are known for their exceptional flying abilities, and their flight capacity can vary depending on the species. On average, most bat species can fly distances ranging from 20 to 50 km in a single night [21]. This ability, known in some species of the Brazilian Atlantic Forest, may influence the positivity described in this study, even though the state of São Paulo does not have many human and animal cases of infection.

*N. laticaudatus*, also known in Brazil as the velvety free-tailed bat, preferentially inhabits flooded forest areas and urban areas. They are social creatures, live in colonies and have seasonal migratory habits. Their migratory ability can influence the acquisition of the infection, as well as their ability to socialize with other bat species. *A. lituratus*, popularly known as the white-faced bat, inhabits forests and is also adapted to urban areas. This is a frugivorous species and travels long distances during the day, which favors the dissemination of seeds and undoubtedly makes them more susceptible to infection. *E. glaucinus* usually lives in urban areas and interacts with other bat species. It feeds on beetles and flies, which may indicate that triatomines are potential prey. *M. molossus*, or velvety free-tailed bat, is the most prevalent urban species in São Paulo. They are social and form huge colonies. They are distributed throughout the South American continent and feed preferentially on mosquitoes. The low positivity in relation to the total number of species collected may be due to the species’ specific feeding habits. *D. rotundus*, the vampire bat, lives in colonies of hundreds of individuals and is known for its social habits of cleaning and self-cleaning, as well as being widely infested with ectoparasites, mainly ticks. Their low prevalence in relation to the total sampled may be due to the fact that they are found in rural areas with little or no housing, which means that there are no environments conducive to the existence of triatomines [24]. To our knowledge, this is the first study to detect DNA of *T. cruzi* in *N. laticaudatus* and *E. glaucinus*.

In our PCR results, there was no positivity in the spleen or intestine. Some strains show a preferential tropism for macrophages in the spleen, liver and bone marrow, while others are very scarce in these organs. The selection of the best tissue for investigation varies greatly [25].

The sequences detected in this study were compared with other South American sequences targeting *T. cruzi* nuclear DNA, and showed interesting positions in the tree, showing the genetic diversity found in bats. It is worth highlighting the sequences obtained from *E. glaucinus* and *M. molossus* are from two bats collected on the same day by the team. Both showed proximity and genetic similarity according to the phylogenetic analysis.

The highest concentration of bats collected was from the central–western region, and as a result, five of the positive samples were from this region. The other positive sample was detected in the northern coastal region. There are few cases of Chagas disease in São Paulo; however, the health services in the municipalities identified in this study need to be alert to new suspected cases of the disease.

One point to discuss is the COVID-19 pandemic, which occurred during the period when the samples were taken. During that period, the control of Chagas disease was neglected throughout Brazil. At the beginning of the pandemic, health resources were significantly redirected to combat COVID-19, including medical personnel, personal protective equipment and funding. This affected the capacity to diagnose and treat endemic diseases, including Chagas disease. The pandemic has temporarily interrupted health services in many areas, including routine medical consultations, diagnostic tests and epidemiological surveillance programs. In addition, the economic and social impact of the pandemic, such as unemployment and reduced resources for public health programs, may have had indirect effects on the control of Chagas disease [26]. The accuracy and reliability of health indicators are closely linked to the quality of the records, i.e., the precision of the information collected. In situations where health systems were overloaded, with a shortage of health professionals and the impossibility of carrying out laboratory tests for all cases, there may have been an overestimation of COVID-19 diagnoses, mainly related to febrile illnesses of other kinds [10].

More than any other parasitic disease, Chagas disease is closely related to social, economic, political and cultural factors, and efforts to interrupt the transmission of the agent of Chagas disease must consider improvements in housing, basic sanitation and people’s quality of life [27].

## 5. Conclusions

Our study detected 3% of bats positive for *T. cruzi* by PCR of organs. *D. rotundus*, *N. laticaudatus*, *A. lituratus*, *E. glaucinus* and *M. molossus* species were associated with positivity. Fruit bats were statistically associated with positivity for *T. cruzi*. To our knowledge, this study detected *T. cruzi* for the first time in bats from São Paulo state and in *N. laticaudatus* and *E. glaucinus* species. Brazilian health agencies need to consider bats in their surveillance of Chagas disease.

## Figures and Tables

**Table 1:** Statistical associations between bat demographic variables and *Trypanosoma cruzi* positivity in the Brazilian Atlantic Forest, São Paulo state (2020–2022).

Variables	*T. cruzi* Positive	*T. cruzi* Negative	OR (95% CI)	*p* Value	Total Population
Eating habit				0.0285	
Frugivorous	1 (25.0%)	3 (75.0%)	1.0 (ref)		4
Insectivorous	3 (3.1%)	95 (96.9%)	10.5 (0.83–133.6)		98
Haematophagous	2 (2.0%)	99 (98.0%)	16.5 (1.15–236.2)		101
Sex				0.4283	
Female	4 (3.9%)	99 (96.1%)	1.0 (ref)		103
Male	2 (2.0%)	98 (98.0%)	1.97 (0.35–11.1)		100
Collection point				0.7342	
Occupied houses	4 (3.5%)	111 (96.5%)	1.0 (ref)		115
Manholes	1 (2.9%)	34 (97.1%)	1.23 (0.13–11.3)		35
Tunnels	1 (7.7%)	12 (92.3%)	0.43 (0.04–4.19)		13
Abandoned houses	0 (0.0%)	38 (100.0%)	3.10 (0.16–59.1)		38
Cave	0 (0.0%)	2 (100.0%)	0.20 (0.01–4.85)		2
Year				0.3867	
2020	1 (7.1%)	13 (92.9%)	1.0 (ref)		14
2021	3 (2.0%)	146 (98.0%)	3.74 (0.36–38.6)		149
2022	2 (5.0%)	38 (95.0%)	1.46 (0.12–17.5)		40
Area				0.4141	
Urban	4 (3.9%)	98 (96.1%)	1.0 (ref)		102
Rural	2 (2.0%)	99 (98.0%)	2.02 (0.36–11.3)		101

*p*-value in bold indicates statistically significant association (*p* > 0.05). 1.0 (ref.): reference category. OR (95% CI): odds ratio (95% Confidence Interval).

**Table 2:** DNA sequencing analysis of the PCR product with specific primers for a region of *Trypanosoma cruzi* nuclear DNA and characteristics of positive samples.

Bat Sample	Bat Specie	Tissue	Bat Sex	Score	Query Coverage	Closest GenBank Match
OR736031	*A. lituratus*	Kidney	Female	279	100%	98.12% (XM800623.1)
OR736032	*E. glaucinus*	Heart	Female	276	96%	98.72% (AY520070.1)
OR736033	*M. molossus*	Heart	Female	246	100%	97.39% (ON872176.1)
OR736034	*N. laticaudatus*	Kidney	Male	261	98%	98.54% (AY520077.1)
OR736035	*D. rotundus*	Heart	Male	237	95%	98.55% (DQ914519.1)
OR736036	*D. rotundus*	Heart	Female	265	100%	98.67% (DQ914484.1)

## Data Availability

Data are contained within the article.
